# The Local Application of Salicylates

**Published:** 1911-07

**Authors:** W. Essex Wynter


### ABSTRACTS

### The Local Application of Salicylates

By W. ESSEX WYNTER, M.D., B.S., F.R.C.P., F.R.C.S.

(From *Folia Therapeutica*, October, 1910)

There are many indications which show that the local application of soluble salicylate, more particularly the salicylate of methyl, may be more efficacious, especially in local rheumatic disorders, than the more usual form of administration by the mouth.

As an instance there may be cited the case of two youths, now in the Middlesex Hospital, who developed signs of pericarditis while convalescing from an attack of rheumatic polyarthritis, and still taking salicylate of soda, which subsided in three days under the application of salicylate of methyl to the precordia. In connection with pericarditis, attention should be drawn to a very important indication, which seems to have been hitherto overlooked, as it is not mentioned in the published accounts of the disease. This is loss of abdominal respiratory movement. The association is so constant that on the one hand this sign should always lead to examination of the pericardium, even when there is nothing else to call attention to a morbid state of that sac, and, on the other hand, when such respiratory movement is definitely present doubtful indications of pericarditis may be disregarded. A very striking instance of this connection between loss of abdominal movement and pericarditis is furnished in the case of a friend's butler, who was seized with acute epigastric pain while waiting at dinner. Absence of abdominal movement was so conspicuous that the man was thought to have a perforated gastric ulcer. Other signs being absent, and the pain diminishing with rest in bed, this view was abandoned, and on the following day, feeling much better, the man got up and went out of his own accord. The pain, however, returned with increased severity, and on further examination a pericardial rub was obvious on the third day. He was admitted to hospital, and made a good recovery under treatment by salicylate of methyl locally, but for quite a fortnight there was no return of abdominal movement.

As a sequel to ordinary rheumatic polyarthritis, it is not unusual to find pain, with stiffness and even swelling persisting in some one joint, after the general fever has subsided. In such cases the local application of salicylates appears often more efficacious than the continued administration by the mouth, and offers the advantage that the curative effect can be thus maintained, without causing the nausea, prostration, and deafness which are liable to attend the former when unduly prolonged. Many cases of sore throat, faucial injection, and follicular or parenchymatous tonsillitis commonly attributed to rheumatism respond rapidly to a compress extending to the angle of the jaw and composed either of the ointment of gaultherium or of a hot fomentation to which a drachm of the oil has been added.

The widest application, however, of this mode of treatment occurs in the various aspects of fibrositis, due to subacute rheumatism affecting nerves, muscles, aponeuroses, and bursæ. Sciatica, brachial neuritis, facial paralysis, and other varieties of local trouble due to rheumatic changes in nerve sheaths usually respond rapidly to the continuous application of the ointment in severe cases, or its intermittent inunction in mild ones, without much local tenderness. Muscular rheumatism, stiff neck, pleurodynia, lumbago, and tenosynovitis, mostly in young people, disappear rapidly under the influence of rubbing with one of the liquid preparations.

The usual influence of salicylate is not, however, restricted to rheumatism, for as sodium salicylate internally is of service in acute stages of gout in reducing fever and pain, so the local application of methyl salicylate is one of the best curative agents for acute gout, particularly in one part, such as the foot. A hot fomentation containing a drachm of gaultherium oil, or one of its preparations with menthol, is the best thing to apply.

After enumerating these many conditions in which the local application of salicylates is of service, it only remains to mention the preparations which are available. The stock remedy is an ointment composed of two drachms of olei gaultherii in an ounce of lanoline, with the addition of fifteen grains of menthol, where an anesthetic effect is required. Antiphlogistine serves as a useful poultice, ready to hand, and containing sufficient methyl salicylate to be effective. A mixture of methyl salicylate with menthol made into a thin ointment is sold in collapsible tubes as anesthol, a convenient remedy for inunction in mild affections, especially chilblains. The liquid preparations are mesotan, Kasemol, and Betul oil. Kasemol, a vinegar-like fluid, is one of the least objectionable in regard to odor and absence of grease; mesotan is similar, but irritating, and best used as a liniment with an equal quantity of olive oil. Betul oil is nearly pure methyl salicylate.

